# Inflammatory Signatures and Biological Markers in Platelet-Rich Plasma Therapy for Hair Regrowth: A Comprehensive Narrative Analysis

**DOI:** 10.3390/diagnostics15091123

**Published:** 2025-04-28

**Authors:** Adelina Vrapcea, Cătălina Gabriela Pisoschi, Eleonora Daniela Ciupeanu-Calugaru, Emil-Tiberius Traşcă, Cristina Violeta Tutunaru, Patricia-Mihaela Rădulescu, Dumitru Rădulescu

**Affiliations:** 1 Doctoral School, University of Medicine and Pharmacy of Craiova, 200585 Craiova, Romania; adelinavrapcea@yahoo.com; 2 Biochemistry Department, Faculty of Pharmacy, University of Medicine and Pharmacy of Craiova, 200585 Craiova, Romania; catalina.pisoschi@umfcv.ro; 3 Department of Biology and Environmental Engineering, University of Craiova, 200585 Craiova, Romania; 4 Department of Surgery, The Military Emergency Clinical Hospital ‘Dr. Stefan Odobleja’, University of Medicine and Pharmacy of Craiova, 200349 Craiova, Romania; dr_radulescu_dumitru@yahoo.com; 5 Department of Dermatology, University of Pharmacy and Medicine Craiova, 200349 Craiova, Romania; cristina.tutunaru@umfcv.ro; 6 Department of Pneumology, University of Pharmacy and Medicine Craiova, 200349 Craiova, Romania; paty_miha@yahoo.com

**Keywords:** platelet-rich plasma (PRP), alopecia, growth factors, inflammation, trichoscopy, combination therapies, precision medicine

## Abstract

**Context:** Hair loss (alopecia) presents both aesthetic and psychological challenges, significantly impacting quality of life. Platelet-rich plasma (PRP) therapy has gained prominence due to its ability to deliver growth factors and modulate local inflammation. However, uncertainties remain regarding the mechanisms through which systemic inflammation, oxidative stress, and coagulation factors influence PRP’s efficacy. **Objectives:** This narrative review explores the impact of inflammatory biomarkers (e.g., NLR, PLR, IL-6, TNF-α) and growth factors (VEGF, TGF-β, FGF) on hair regeneration in PRP therapy. It discusses how oxidative stress and vitamin status (B12, D, folate) correlate with therapeutic success. Additionally, it examines the PRP preparation protocols and combined approaches (microneedling, minoxidil, LLLT) that may amplify clinical responses. **Results:** The synthesized data highlight that elevated systemic inflammation (increased NLR/PLR values) can limit PRP’s effectiveness, while the regulation of inflammation and optimization of antioxidant status can enhance hair density and thickness. Integrating vitamins and an anti-inflammatory diet into the therapeutic protocol is associated with more stable hair growth and reduced adverse reactions. The variability in PRP’s preparation and activation methods remains a major obstacle, underscoring the need for standardization. **Conclusions:** Integrating inflammatory biomarkers with oxidative stress indicators provides fresh insights for tailoring PRP therapies in alopecia. Multimodal treatment strategies combined with collaborative multicenter studies—in which biological markers are embedded within rigorous protocols—could establish standardized methodologies and significantly enhance the treatment success.

## 1. Introduction

### 1.1. The Context of Hair Loss and the Importance of Regenerative Treatments

Hair loss, broadly referred to as alopecia, impacts a significant portion of the global population, exerting complex effects both aesthetically and psychologically. Patients diagnosed with alopecia areata in particular may experience elevated levels of anxiety and depression, as evidenced by studies reporting a high prevalence of these disorders among individuals with moderate to severe forms of the condition [1]. While initially perceived as a purely cosmetic concern, hair loss can provoke intense emotional responses, disrupting social adaptation and interpersonal relationships.

From a gender perspective, the psychological repercussions of alopecia are often more pronounced in women, who frequently report a negative body image and feelings of social vulnerability [2]. Although men might downplay its aesthetic impact in certain contexts, they too can suffer significant distress, especially when balding occurs at a young age. Research indicates that androgenetic alopecia in women is associated with substantial emotional stress, sometimes exceeding that experienced by men, as their hair is closely linked to their femininity and attractiveness [2].

Moreover, quality of life is significantly compromised across multiple dimensions, including self-esteem, perceived social stigma, and emotional functioning, for those experiencing hair loss [3]. Conditions such as alopecia areata and androgenetic alopecia can lead to negative emotional responses, including social withdrawal or the avoidance of situations where individuals feel exposed, such as social gatherings. In adolescents and young adults, academic pressures and familial expectations can heighten their anxiety related to hair loss, leading to additional challenges in peer group integration [4].

Even though younger patients may maintain higher self-esteem, they can still experience elevated levels of social anxiety compared to older adults [5]. Consequently, gender and age differences are crucial considerations in the therapeutic approaches, necessitating tailored psychological and medical management strategies. For women, the psychosocial burden intensifies significantly when alopecia affects their eyebrows, potentially leading to feelings of “dehumanization” or perceptions of premature aging [3].

Patients often adopt coping strategies such as wearing wigs, selecting specific hairstyles, using scarves or makeup, or applying micropigmentation to bald areas. While these measures can provide temporary psychological relief, they may also become sources of anxiety themselves (e.g., fear of wig slippage, concern over others’ reactions, or the revelation of a “hidden problem”) [6]. Additionally, for individuals experiencing extensive hair loss, such as those with universal alopecia areata, significant events (e.g., weddings, job interviews) can exacerbate psychological stress.

Given these psychological impacts, there is a clear need for therapeutic approaches that extend beyond mere aesthetic correction. Integrating psychological support into treatment regimens is essential to mitigate or prevent the long-term amplification of negative effects [1,2,3]. Psychological counseling and therapies aimed at enhancing self-esteem can complement medical treatments, improving patient compliance and reducing the likelihood of treatment discontinuation.

Beyond their role in physical healing, hair regeneration therapies can significantly restore patients’ confidence in their body image and reestablish psychological equilibrium. Therefore, combining medical treatments with psychological support is imperative the comprehensive management of alopecia, contributing to an enhanced quality of life and social reintegration for patients.

Platelet-rich plasma (PRP) therapy has emerged as a compelling option for hair loss due to its minimally invasive nature, excellent safety profile, and unique regenerative capacity, arising from a high concentration of platelet-derived growth factors and bioactive molecules. Unlike pharmacologic agents such as minoxidil or finasteride, PRP harnesses the body’s own reparative processes by enhancing follicular angiogenesis (via VEGF), reducing local inflammation (via IL-6 and TNF-α inhibition), and stimulating dermal papilla cell proliferation (via PDGF, FGF, and IGF). Multiple studies and systematic reviews support its efficacy; for example, a 2024 meta-analysis of randomized trials in women found that PRP significantly increased their hair density and thickness while maintaining a low rate of adverse effects and high patient satisfaction [7]. Likewise, a 2023 meta-analysis involving 864 patients concluded that activated PRP (A-PRP) is both safe and effective for enhancing hair growth and terminal hair density in alopecia [8]. These findings align with earlier clinical trials demonstrating marked improvements in hair follicle units and overall satisfaction, especially in patients unresponsive to conventional medications [9].

### 1.2. Platelet-Rich Plasma (PRP) as a Therapy for Hair Growth

Platelet-rich plasma (PRP) therapy has emerged as a contemporary therapeutic option for treating various forms of alopecia, including alopecia areata and androgenetic alopecia [10]. PRP is derived from the patient’s own blood, which is centrifuged to isolate plasma enriched with platelets from the other blood components. Upon activation, platelets release a spectrum of growth factors (such as VEGF, PDGF, TGF-β, and FGF) that play crucial roles in tissue regeneration and the modulation of inflammatory processes [11].

The preparation of PRP can vary considerably depending on the centrifugation protocols. Some studies suggest that double centrifugation enhances the platelet recovery and growth factor concentration [12], while others utilize simpler yet effective methods [13]. Certain commercial systems for PRP extraction—such as the GPS III Platelet Concentration System (Zimmer Biomet, Warsaw, IN, USA) and the Magellan Autologous Platelet Separator System (Arteriocyte Medical Systems, Inc., Cleveland, OH, USA) provide standardized PRP products, although their performance may differ from that of other devices (e.g., the Selphyl PRFM System; Aesthetic Factors, LLC, Wayne, NJ, USA) [14]. Additionally, the inclusion of leukocytes in PRP remains controversial: some researchers argue that leukocyte-rich PRP can aid in combating infections, whereas others claim that leukocytes may exacerbate inflammation, which is undesirable in certain contexts [15].

The standard recommendations typically involve centrifugation parameters of 100–300 g for 5–10 min during the initial spin, followed by 400–700 g for 10–17 min, aiming to achieve an optimal platelet concentration of 1–1.5 million platelets/µL [16]. Consequently, each medical center can tailor its protocols based on the available equipment and the expertise of the medical team.

Clinically, numerous randomized studies have demonstrated the efficacy of PRP in reducing the rate of hair loss, increasing hair density, and enhancing hair thickness [17,18]. In a controlled study with 25 patients, PRP significantly increased the percentage of hairs in the anagen phase compared to that in the placebo [17]. Additionally, in women with androgenetic alopecia, PRP treatment resulted in observable improvements both from the patients’ subjective perspectives and in the investigators’ assessments [18]. Further research indicates that activated PRP (using calcium chloride or other agents) may elicit a more rapid response, sometimes leading to significant increases in new hair density [19,20].

A recent meta-analysis concluded that PRP, compared to a placebo, yielded significant improvements for patients with androgenetic alopecia and possessed a favorable safety profile, with minimal adverse reactions such as erythema and local discomfort [21]. This overall efficacy, combined with the procedure’s minimally invasive nature and relatively few side effects, accounts for the growing interest in PRP among dermatologists and patients alike.

### 1.3. The Importance of Biological and Inflammatory Markers in PRP Therapy

Inflammation plays a well-documented role in the pathogenesis of various alopecias (areata, androgenetic, scarring), with numerous studies linking immune system activation to structural damage to the hair follicles [22]. In alopecia areata, for instance, intense lymphocytic infiltrates (including CD8+ T cells, eosinophils, and mononuclear cells) correlate with the severity of hair loss, and elevated serum IgE levels may indicate localized inflammatory responses [22]. These findings suggest that autoimmune mechanisms and cytokine imbalances are pivotal in disease progression.

Conversely, certain genetic factors, such as polymorphisms in IL-1α, may predispose individuals to inflammation and influence their response to PRP therapy [20]. The complexity increases in scarring alopecias (e.g., lichen planopilaris, discoid lupus erythematosus), where chronic inflammation and lichenoid reactions result in irreversible follicular destruction [23]. Additionally, in androgenetic alopecia, primarily driven by hormonal factors (dihydrotestosterone), perifollicular inflammation can accelerate hair shaft miniaturization [24]. Stress further exacerbates the condition by increasing the secretion of pro-inflammatory mediators, promoting the transition to the catagen phase and intensifying hair loss [25].

In this context, the growth factors provided by PRP (VEGF, TGF-β, FGF) have a dual function: (a) supporting regeneration and cellular proliferation through enhanced vascularization and (b) modulating local inflammation [26]. Studies have demonstrated that the levels of VEGF, TGF-β, and FGF are significantly elevated in PRP-treated plasma compared to those in control groups [27]. The different PRP preparation methods affect the concentrations of these growth factors, with activated PRP typically providing higher levels of VEGF and TGF-β [28]. The substantial increase in VEGF, FGF, and TGF-β following PRP administration stimulates tissue repair, reduces inflammation in the affected areas, and promotes the reentry of hair follicles into the anagen phase [28]. Furthermore, TGF-β may serve as a potential biomarker for assessing therapeutic efficacy, with studies indicating correlations between serum levels and the response rate to PRP therapy [29].

Thus, investigating inflammatory and biological markers offers valuable insights into the variability in the responses to PRP therapy. Some patients may benefit from combined therapeutic approaches, such as anti-inflammatory interventions, evaluation of genetic polymorphisms, and regulation of overall pro-inflammatory status, either before or alongside PRP administration. This integrated approach could facilitate personalized therapy, enhancing the likelihood of successful outcomes and reducing recovery times.

## 2. Materials and Methods

For this narrative review, an exhaustive search was conducted in major medical databases, including PubMed (National Library of Medicine, Bethesda, MD, USA; accessed 15 October 2024), Web of Science (Clarivate Analytics, Philadelphia, PA, USA; accessed 15 October 2024), and Embase (Elsevier, Amsterdam, The Netherlands; accessed 15 October 2024). The search utilized relevant keywords such as “PRP therapy”, “platelet-rich plasma”, “alopecia”, “hair loss”, “inflammatory markers”, and “growth factors”. The search strategy incorporated combinations of these terms to ensure comprehensive coverage of the available literature on PRP therapy in the context of alopecias.

The analysis included original studies, randomized clinical trials, meta-analyses, and narrative reviews that explored the relationship between PRP therapy, inflammatory markers, and hair regeneration. The inclusion criteria targeted articles published in English in recognized specialty journals that provided detailed information on the PRP preparation methods and evaluated the inflammatory parameters and clinical outcomes in the treatment of alopecia.

Excluded from this review were articles with unclear methodological designs, isolated case reports, or studies lacking sufficient information on biological parameters and inflammation. Additionally, systematic reviews or meta-analyses that did not offer a specific synthesis regarding the relationship between PRP and inflammatory markers in the context of hair regeneration were excluded.

The data extracted from the selected studies were organized thematically, addressing the following main categories:

- The physiopathological mechanisms through which PRP influences hair follicle regeneration;
- The role of inflammation in the pathogenesis of alopecias and how PRP contributes to its reduction;
- The specific biological markers involved in the response to PRP therapy;
- Management perspectives and strategies for optimizing PRP therapy through the integration of inflammatory markers and complementary technologies.

This structured approach facilitated a detailed and comprehensive analysis of the existing literature, enabling the identification of research gaps and the proposal of future research directions in the field of PRP therapy for alopecias.

## 3. Relevant Physiopathological Mechanisms in PRP Therapy and Hair Loss

PRP therapy targets the core pathogenic mechanism in alopecia—chronic local inflammation that disrupts hair follicle regeneration—by simultaneously enhancing local vascularization, modulating inflammatory responses, and stimulating key follicular cells.

Upon platelet activation, PRP releases a spectrum of growth factors, including VEGF, TGF-β, FGF, and PDGF. VEGF (Vascular Endothelial Growth Factor) is crucial for promoting angiogenesis; it increases perifollicular capillary density and enhances the delivery of oxygen and nutrients, which is essential for maintaining the anagen (growth) phase of the hair cycle [30]. Enhanced vascularization not only supplies energy to the follicles but also creates a more favorable microenvironment for their regeneration.

TGF-β (Transforming Growth Factor-beta) plays a dual role. While its isoforms TGF-β1 and TGF-β2 can trigger apoptotic pathways and initiate the catagen phase under certain conditions (thus potentially contributing to follicular regression) [31,32], the optimal levels support tissue regeneration by stimulating extracellular matrix synthesis and modulating inflammation [33]. The balance of TGF-β signaling is therefore critical; inhibiting excessive TGF-β activity can delay follicular degradation.

FGFs (Fibroblast Growth Factors)—notably FGF-7, FGF-10, and FGF-18—along with PDGF (Platelet-Derived Growth Factor) stimulate the proliferation and differentiation of the dermal papilla cells and support the inductive properties of the follicular matrix. These factors maintain the regenerative potential of bulbar stem cells, ensuring sustained hair growth [34,35,36,37]. Moreover, studies comparing PRP preparation methods have revealed that protocols enhancing the platelet concentration and pre-activation (e.g., using calcium chloride) yield higher levels of these growth factors, thus amplifying their regenerative and anti-inflammatory effects [29,38,39].

A key aspect of PRP’s efficacy is its ability to counteract chronic local inflammation—a major pathogenic factor in both androgenetic alopecia and alopecia areata. Chronic inflammation leads to the increased local expression of pro-inflammatory cytokines, such as IL-6 and TNF-α, which inhibit hair follicle regeneration by impairing the function of follicular stem cells and promoting follicular miniaturization [28,40]. PRP administration reduces the levels of these cytokines, thereby restoring follicular homeostasis and enhancing the regenerative environment.

In addition to cytokine modulation, PRP contributes to extracellular matrix (ECM) remodeling by stimulating the synthesis of collagen and elastin while concurrently downregulating the matrix metalloproteinases (MMPs) that degrade components of the ECM under chronic inflammation [41,42,43,44,45,46,47]. This ECM stabilization reinforces the structural integrity of the scalp and supports follicle health.

Furthermore, PRP exhibits synergistic effects when combined with other treatment modalities—such as minoxidil, low-level laser therapy (LLLT), or microneedling—resulting in improved local penetration of growth factors, enhanced blood flow, and a further reduction in inflammation [48].

In summary, PRP corrects the primary pathogenic mechanism in alopecia—chronic local inflammation—through the following processes:

- Increasing local vascularization through VEGF, thereby sustaining the anagen phase;
- Reducing pro-inflammatory cytokines (IL-6, TNF-α) to restore a balanced immune environment;
- Stimulating the dermal papilla and follicular matrix cells via FGF and PDGF, which supports follicular regeneration.

**Figure 1:**
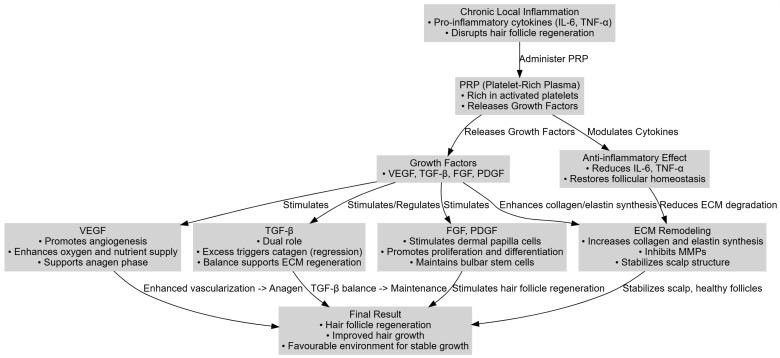
PRP mechanism flowchart.

By effectively modulating these processes, PRP creates a regenerative microenvironment that not only counteracts the deleterious effects of chronic inflammation but also promotes stable hair regrowth and improved scalp health (Figure 1).

## 4. Inflammatory and Biological Markers Associated with Response to PRP

### 4.1. The Indicators Proposed in the Literature

A variety of cellular ratios, including the neutrophil–lymphocyte ratio (NLR), the platelet–lymphocyte ratio (PLR), and the Monocyte–Lymphocyte Ratio (MLR), are increasingly utilized in research to quantify systemic inflammation levels. Systemic inflammation is a significant factor in the progression of numerous diseases and can directly or indirectly influence the response to regenerative therapies such as platelet-rich plasma (PRP).

Numerous studies have explored the association between the NLR and therapeutic response. For example, Jin et al. determined threshold values of 2.464 for the NLR and 106.3 for the PLR, correlating higher levels with better response rates [46]. In oncology, Graziano et al. highlighted that an NLR of 2.42 and a PLR of 104.47 can predict a complete pathological response to neoadjuvant therapy [47]. In the context of alopecia, although the volume of research is not as extensive, the general principle remains valid: a moderate level of systemic inflammation, reflected by a lower NLR, may suggest a more favorable immunological environment for regeneration.

The PLR provides additional insight into immune system reactivity, as platelets also play a role in inflammatory processes. Studies conducted by Gou et al. in gastric cancer and Darzikolaee et al. in colorectal cancer demonstrated threshold values for the NLR and the PLR associated with better survival and more effective therapeutic responses [49,50]. Although these results originate from the field of oncology, they suggest, by analogy, that a lower PLR could indicate a less aggressive inflammatory status and consequently a greater regenerative capacity in the context of PRP therapies.

While less investigated compared to the NLR and PLR, the MLR can provide insights into the state of chronic inflammation, as the monocytes are involved in tissue repair mechanisms, as well as autoimmune processes and excessive fibrosis. Monitoring the MLR in combination with the NLR and PLR can enhance the accuracy of assessing overall inflammatory status.

In addition to these ratios, other parameters such as the Basophil–Lymphocyte Ratio (BLR), the Eosinophil–Lymphocyte Ratio (ELR), the Systemic Immune-Inflammation Index (SII), and the Systemic Inflammation Response Index (SIRI) have also been mentioned. Although there are no studies directly linking the ELR and the SIRI to the success of PRP in alopecia, experiences from other medical specialties indicate that these indices can reflect the severity of systemic inflammation [51]. Integrating them into future research on PRP could reveal new correlations between the degree of systemic inflammation and the rate of hair regeneration.

In conclusion, the threshold values for the NLR and PLR (generally below 2.5–3.0 for the NLR and below 150–200 for the PLR, depending on the specific pathology) may suggest a controlled inflammatory status. By extension, in patients with alopecia presenting low values for these indicators, PRP may have increased chances of producing beneficial outcomes.

While these ratios serve as indicators of systemic inflammation, elevated NLRs and PLRs do not directly alter the production or activity of growth factors in PRP; rather, they reflect a host environment characterized by increased levels of pro-inflammatory cytokines such as IL-6 and TNF-α [52]. This pro-inflammatory milieu can impair the responsiveness of the hair follicles to the regenerative signals provided by PRP—even when the preparation contains the optimal concentrations of PDGF, VEGF, and TGF-β [11]. In this regard, a controlled inflammatory status—reflected by a lower NLR (generally below 2.5–3.0) and PLR (below 150–200)—appears essential to maximizing the therapeutic benefits of PRP in alopecia, as suggested by studies in oncology and analogous research [46,47,49,50,51]. Consequently, pre-treatment interventions aimed at reducing systemic inflammation may enhance clinical outcomes, making these ratios valuable predictive biomarkers for PRP’s efficacy [20].

### 4.2. Interpretation and Clinical Relevance

In medical practice, the accurate interpretation of the NLR and PLR must consider the patient’s history, comorbidities, and other inflammatory factors such as C-reactive protein (CRP) and fibrinogen. Elevated levels of the NLR and the PLR above the threshold values may indicate a pro-inflammatory environment, which has been associated with poorer therapeutic responses in various pathologies [53].

High NLRs and PLRs are indicative of a poor therapeutic response. In a study on immunologic therapy, Suh et al. observed that an NLR ≥ 5 post-treatment was associated with an unfavorable prognosis [53]. In the context of PRP for alopecia, it can be speculated that increased systemic inflammation might counterbalance the regenerative effects of the growth factors, limiting the follicles’ ability to initiate the anagen phase.

Conversely, low NLRs and PLRs suggest a potential favorable response. Basher et al. demonstrated that patients responding to immunotherapy exhibited significant decreases in their NLRs and PLRs, suggesting a reduction in systemic inflammation [54]. In the case of PRP, reduced inflammation would create a more receptive microenvironment for the growth factors released by the platelets. Furthermore, Ohlendorf et al. correlated decreased CRP levels and an NLR below 2.5 with better therapeutic responses in various targeted therapies [55]. Specifically, in patients with alopecia presenting a relatively low pro-inflammatory status, the effect of PRP could be enhanced.

Thus, monitoring patients’ inflammatory ratios before, during, and after PRP treatment provides valuable information about the evolution of their inflammatory status and the potential success of the therapy. An initially high NLR/PLR value that remains elevated or even increases after the initial sessions of PRP therapy may indicate that inflammation is not controlled and that additional interventions, such as anti-inflammatory therapy or more comprehensive evaluations to identify underlying conditions, are necessary. Conversely, a significant decrease in these indices, alongside trichoscopic improvements, can confirm the effectiveness of the chosen therapeutic strategy.

### 4.3. Other Biological Markers

In addition to inflammatory ratios, the levels of certain pro-inflammatory cytokines and coagulation factors used in PRP preparation can directly influence the quality and stability of the final product, as well as the clinical response.

Interleukin-6 (IL-6) is considered a central marker of chronic inflammation and the progression of various types of alopecia [56]. The elevated presence of IL-6 in the follicular area can block the anagen phase and accelerate hair loss. Tabara et al. demonstrated that patients with alopecia areata exhibit significantly higher serum levels of IL-6, underscoring the pro-inflammatory role of this cytokine [56]. Interventions that reduce IL-6, such as the administration of PRP containing growth factors with anti-inflammatory effects, can restore balance in the follicular microenvironment [57].

Interleukin-1 (IL-1) and Tumor Necrosis Factor-alpha (TNF-α) are involved in inflammatory cascades that can exacerbate alopecia. The overproduction of IL-1β promotes follicular structure degradation, while TNF-α can suppress the proliferation of the dermal papilla cells. Their reduction following PRP treatment, as documented in some studies, suggests the potential anti-inflammatory role of platelet-derived growth factors [52,58].

The PRP preparation process involves either immediate platelet activation post-collection using chemical agents such as calcium chloride, bovine thrombin, or photostimulation or its natural, slow activation during its administration [39]. Textor and Tablin demonstrated that activation using calcium chloride results in a superior release of Platelet-Derived Growth Factor (PDGF), and the resulting PRP contains higher levels of VEGF and TGF-β [39]. Weibrich et al. and Cole et al. confirmed that activating PRP significantly increases the growth factor concentrations, maximizing its regenerative potential [27,28].

Another activation technique mentioned by Irmak et al. involves photostimulation with polychromatic light (in the spectrum of 600–1200 nm), which facilitates the prolonged release of growth factors, maintaining beneficial effects for an extended period of up to 28 days [59]. Clinically, this activation method may be useful when a long-lasting effect of PRP and continuous stimulation of the hair follicles are desired.

Thus, IL-6, IL-1, and TNF-α represent a crucial set of pro-inflammatory markers that can be measured to assess alopecia’s severity and the response to PRP treatment. Simultaneously, coagulation factors and PRP activation techniques determine the optimal release of growth factors, significantly influencing therapeutic efficacy. By personalizing the activation method—using calcium chloride, thrombin, or photostimulation—and monitoring pro-inflammatory cytokines, clinicians can adjust the treatment protocols to maximize the outcomes for each individual patient.

## 5. The Impact of PRP on Oxidative Stress and Vitamins

PRP therapy has emerged as a prominent treatment for alopecia, not only due to its direct regenerative effects on the hair follicles but also because of its influence on oxidative stress and vitamin status, both of which are crucial for hair health. This section succinctly examines how PRP interacts with the antioxidant system and essential vitamins to mitigate pathological conditions associated with hair loss.

PRP administration enhances the activity of antioxidant enzymes such as superoxide dismutase (SOD) and glutathione peroxidase (GPx), thereby mitigating oxidative stress [60]. By releasing growth factors and bioactive molecules, PRP boosts the antioxidant defense, protecting hair follicle cells from oxidative damage and supporting healthy hair growth.

PRP reduces the levels of lipid peroxides and protein carbonyls, key markers of oxidative stress, thereby safeguarding the structural integrity of the hair follicles [61]. This protective effect helps maintain cellular membranes and protein structures, fostering an environment conducive to hair regeneration and long-term scalp health.

PRP increases the levels of reduced glutathione (GSH) and the activity of glutathione peroxidase (GPx), enhancing antioxidant homeostasis [62]. This restoration of redox balance in the hair follicle cells prevents their deterioration and promotes healthy hair growth, contributing to the overall regenerative process.

Adequate levels of vitamins B12, D, and folate are essential for maximizing PRP’s therapeutic effects [63]. These vitamins play critical roles in DNA synthesis, cellular regeneration, and immune regulation. Deficiencies can exacerbate inflammation and oxidative stress, limiting PRP’s efficacy. Thus, integrating vitamin supplementation and maintaining a balanced diet can enhance the treatment outcomes and prevent the recurrence of hair loss.

Diets rich in antioxidants (vitamins C and E) and anti-inflammatory microelements optimize the biological environment for PRP therapy [64]. Combining PRP with nutritional strategies, including omega-3 and omega-6 fatty acids, zinc, selenium, and nutraceutical supplements, can reduce inflammation further and support hair health. These integrated approaches amplify PRP’s regenerative potential, leading to more effective and sustained hair growth.

In conclusion, PRP significantly impacts oxidative stress and vitamin status, essential factors in managing alopecia. By enhancing antioxidant enzyme activity, reducing oxidative markers, increasing glutathione levels, and optimizing vitamin status, PRP creates a favorable environment for hair regeneration. Additionally, combining PRP with nutritional interventions can amplify its benefits, offering improved outcomes and robust hair health for patients.

## 6. Clinical and Paraclinical Evaluations of PRP’s Efficacy

A comprehensive assessment of the response to PRP therapy involves both objective methods, which directly measure follicular parameters and scalp structure, and subjective methods, which quantify a patient’s perception of their progress and quality of life. These evaluations are essential for the rigorous documentation of PRP’s efficacy and for adjusting the therapeutic protocols based on outcomes.

### 6.1. Evaluation Methods

#### 6.1.1. Trichoscopy

Trichoscopy is one of the most widely utilized evaluation techniques in studies on alopecia and the response to regenerative therapies. This non-invasive method allows for a detailed analysis of hair strand characteristics and scalp condition, providing insights into hair density, hair thickness, the terminal-to-vellus ratio (T:V), and signs of inflammation such as scaling, hyperkeratosis, the presence of black dots, and exclamation mark hairs in alopecia areata [48].

Kaur et al. highlighted significant improvements in hair density and the T:V ratio in patients with androgenetic alopecia after PRP administration [48]. Additionally, periodic trichoscopic evaluations enabled the real-time detection of reduced miniaturized hairs and an increased proportion of terminal hairs. In a randomized study, Rodrigues et al. utilized TrichoScan to measure the total hair count, density, and the proportion of hairs in the anagen phase, reporting significant increases compared to these values in the control group [11].

A distinct technical approach involves comparing different injection methods, such as Dermapen and pinpoint injections, as conducted by Ozcan et al. [65] Their findings emphasize that Dermapen ensures a uniform distribution of PRP across the scalp’s surface, leading to more consistent improvements in hair density and terminal hair thickness [65]. Through trichoscopy performed with TrichoScan^®^ software (version not specified in Özcan et al. [65]; Trichosys GmbH, Munich, Germany), clinicians can quantify the progress achieved through PRP at each visit, detecting even subtle changes that might be missed during a simple visual examination of the scalp.

#### 6.1.2. Standardized Photography and Objective Measurements

Photographs taken under standardized conditions—at a fixed angle, with a consistent lighting intensity, and using identical patient positioning—serve as a convenient and valuable tool for documenting results. This approach allows for a comparison of the scalp’s appearance before and after treatment, the creation of an image archive for later review by other specialists, and objective discussions with patients about their progress and potential treatment limitations.

Gentile et al. utilized standardized photographs to evaluate parameters such as hair density and thickness, highlighting consistent improvements following PRP administration [66]. Another highly useful technique is computerized phototrichograms, as applied in studies by Dhurat and Saraogi, which provide automated information on hair count and diameter, thereby reducing the possibility of human error [67].

The standardized protocols proposed by Takwale et al. include the use of neutral backgrounds, maintaining the same distance between the scalp and the camera, and consistent lighting settings (Institute of Medical Illustrators, 2024) [68]. These precautions ensure high comparability between images captured at different time points. Global photography, as described by Verma et al., offers an overview of the scalp and is used to monitor progress in the frontal, parietal, or vertex areas [69].

Combining trichoscopy with standardized photography facilitates a comprehensive evaluation. Dhurat and Saraogi emphasize that by assessing both objective parameters and photographic evidence, the outcomes of one modality can validate those of the other, thereby enhancing the reliability of conclusions about PRP’s efficacy [67].

#### 6.1.3. The Use of FotoFinder Technology in Evaluations

FotoFinder is an advanced digital scalp analysis system that combines high-resolution imaging techniques with automated analysis software. This technology enables measurements of hair density and diameter, time-sequential analyses with digital overlays to accurately visualize progress, and the integration of artificial intelligence algorithms for automatic follicle detection.

Kim et al. implemented YOLOv4 and EfficientDet neural networks in FotoFinder systems, achieving high accuracy in analyzing the hair density in captured images [70]. TrichoScan, an integrated module of FotoFinder, has been validated in various clinical studies for automatically measuring hair density and diameter, showing excellent correlation with manual methods [71]. By eliminating operator subjectivity, FotoFinder reduces the margin of error and ensures quality control in evaluations conducted in multicentric or comparative studies [72].

Lee et al. employed similar automated image analysis methods to measure follicular density and average hair diameter, demonstrating very good concordance between their automated and manual results [72]. Gudobba et al. advanced these techniques by developing automatic hair loss quantification algorithms, achieving over 90% accuracy [73].

Although studies have not yet reported a direct correlation between NLR/PLR values and FotoFinder measurements, the potential to integrate these variables remains significant. In future scenarios, digital systems could simultaneously generate data on hair density and correlate it with an inflammatory score, allowing for personalized adjustments to PRP treatment frequency and duration.

### 6.2. Subjective Parameters

#### 6.2.1. Patient Satisfaction

Subjective parameters complement paraclinical methods by providing information about an individual patient’s perception and how objective scalp changes influence their psychological well-being and quality of life.

Standardized questionnaires, such as the Dermatology Life Quality Index (DLQI) or Scalpdex, assess the impact of hair loss on dimensions such as emotions, social functioning, and self-perception [74]. Chernyshov et al. demonstrated that the DLQI significantly correlates with alopecia severity, offering a quantified measure of its social and emotional impact [74].

Other instruments, like the Alopecia Areata Quality of Life Index (AA-QLI), measure the specific effect of hair loss in alopecia areata, while the Children’s Dermatology Life Quality Index (CDLQI) addresses pediatric patients [75,76]. To assess anxiety and depression levels, clinical scales such as the Hospital Anxiety and Depression Scale (HADS) can be used. Additionally, questionnaires specifically designed for women with androgenetic alopecia, such as the Women’s Androgenetic Alopecia Quality of Life (WAA-QOL), track this particular impact on emotional and social dimensions [77].

#### 6.2.2. The Importance of Psychological Counseling and Patient Expectations

Studies indicate that patients’ perception of hair improvements is often filtered by their expectations and emotional states. Psychological interventions, including counseling and psychoeducation, can provide significant benefits in shaping realistic expectations. For example, Meyers et al., using the HAIRDEX scale, observed significant improvements in emotions, social functioning, and self-confidence in patients receiving concurrent psychological counseling [78]. Papakonstantinou et al. noted that well-informed patients who received emotional support reported higher satisfaction with PRP treatment outcomes [79].

Furthermore, Gentile et al. observed that reducing emotional stress through psychological counseling before PRP administration contributes to a better therapeutic response and increased treatment adherence [66]. Rossano et al. suggest that well-counseled patients perceive aesthetic treatments more favorably, even when the objective improvements are subtle, demonstrating the major influence of psychological factors [20].

Therefore, the evaluation of PRP’s efficacy should include detailed paraclinical investigations, such as trichoscopy, standardized photography, and digital systems like FotoFinder, as well as a subjective analysis of quality of life using instruments like the DLQI, Scalpdex, the WAA-QOL, and the HADS. Only by combining these techniques can a comprehensive picture be obtained of how PRP contributes to hair regeneration and enhances patients’ well-being. Additionally, the results of these evaluations aid in refining the protocols, personalizing the administration intervals, and providing appropriate psychological support, thereby increasing the overall efficacy of PRP therapy.

## 7. Perspectives from the Literature and Mechanistic Insights

A comprehensive understanding of the role of PRP in treating alopecia arises from synthesizing the results from numerous clinical and experimental studies, as well as interpreting the mechanisms of its interaction with the systemic and local inflammation. This section consolidates essential conclusions from the specialized literature, explores potential mechanisms for enhancing PRP’s effects, and addresses the current limitations, highlighting the necessity of the future research directions.

### 7.1. A Summary of Key Studies

Most of the available studies report modest to significant improvements in hair density following PRP treatments; however, the results vary notably due to differences in the protocols, such as the number of injections, activation methods, and platelet concentration. For instance, Gentile et al. compared activated versus non-activated PRP and found that the activated variant induced greater increases in hair density, reaching values of +90 ± 6 hairs/cm^2^ compared to +65 ± 5 hairs/cm^2^ with non-activated PRP [66]. Similarly, in another study, Gentile et al. confirmed these benefits, reinforcing the advantages of the activated method [80].

A review of systematic analyses and meta-analyses indicates that PRP significantly enhances hair density and thickness and overall hair condition compared to these values under a placebo [21]. Nonetheless, the degree of the improvement remains variable, influenced by the platelet concentration and type of activation. In a subsequent review, Morkuzu et al. concluded that PRP leads to a notable increase in hair count and thickness but emphasized that the limited number of randomized studies and the lack of standardization reduce the strength of these conclusions [8].

Comparing protocols using different platelet concentrations reveals that higher platelet concentrations generally ensure better effects on hair density and diameter [19]. However, differences in the terminal-to-vellus hair ratio are not consistently statistically significant [81]. Additionally, combining PRP with other technologies, such as microneedling or low-level laser therapy (LLLT), can enhance the treatment success rates further. For example, a study combining PRP with microneedling demonstrated an increase of approximately +81 ± 5 hairs/cm^2^ compared to that in PRP monotherapy [48,82].

Studies investigating the reference values for inflammatory markers (NLR, PLR) in the context of alopecia are still limited. However, it has been proposed that an NLR ≥ 2.2–2.3 and a PLR ≥ 150–200 may correlate with disease severity and a poorer response to certain therapies [83,84,85].

### 7.2. Possible Mechanisms of Interaction

Elevated systemic inflammation can diminish the positive effects of PRP by disrupting the balance mediated by growth factors and promoting catagen processes within the hair follicles. Comparative studies highlight that patients with chronic inflammation (characterized by high NLR/PLR markers and elevated CRP levels) exhibit lower success rates under regenerative treatments [86]. PRP, through the release of growth factors (such as VEGF, TGF-β, and FGF) and its localized anti-inflammatory action, can partially counteract systemic inflammation, facilitating the transition of follicles back into the anagen phase [28].

Combining PRP with other therapies has demonstrated increased efficacy. For instance, minoxidil and LLLT can potentiate PRP’s effects by enhancing scalp microcirculation and directly stimulating the follicular matrix cells [87,88,89]. Additionally, microneedling creates microchannels through the stratum corneum, facilitating the penetration and diffusion of PRP-derived growth factors.

Regulating scalp inflammation remains a critical component. PRP combined with minoxidil and finasteride has been shown to reduce inflammatory infiltrates around the hair follicles and improve regeneration, suggesting a synergistic effect. Understanding the precise ways in which PRP acts on inflammation and tissue regeneration can open avenues for more effective, individualized therapies.

### 7.3. Current Study Limitations and the Path Forward

While the therapeutic potential of PRP in alopecia management is undeniable, the field faces several critical challenges that must be addressed to maximize its efficacy. The lack of standardized preparation protocols for PRP remains a major obstacle. Variations in the centrifugation techniques, platelet concentrations, and activation methods make it difficult to draw firm conclusions about the “optimal” approach. Some commercial kits, such as GPS III and Magellan, yield high platelet concentrations but also increase leukocyte content, which could influence inflammation at the application site [40,90,91,92]. Conversely, systems like Selphyl produce leukocyte-poor PRP, creating further variability in the results [93].

Another limitation stems from the inconsistent relationship between the platelet concentration and clinical outcomes. Although higher platelet counts often yield better results, this is not universally observed, suggesting that additional factors, such as growth factor quality or patient-specific variables, play a role [80].

Moreover, small sample sizes and short follow-up durations in the existing research hinder the ability to assess PRP’s long-term benefits and safety profile. Most studies have involved fewer than 30 participants and tracked the outcomes over a few months, leaving critical gaps in our understanding of the sustainability of PRP’s effects and its efficacy across different patient populations [94,95,96].

The underreporting of biological markers like IL-6, TNF-α, and TGF-β is another significant gap. Without these data, it is challenging to establish a direct connection between PRP’s biological mechanisms and its clinical outcomes. For example, tracking changes in systemic inflammation markers (NLR, PLR) alongside clinical improvements could help refine the patient selection and predict treatment success [97].

Finally, while research on PRP has advanced significantly, there has been limited integration of cutting-edge technologies such as transcriptomics, proteomics, and metabolomics. These multi-omics approaches could shed light on the molecular pathways through which PRP exerts its effects, offering insights into how inflammation, growth factors, and tissue regeneration interact [98]. Additionally, randomized multicenter studies are urgently needed to validate the findings and minimize the biases introduced by single-center research [94,95,96].

## 8. Future Research Directions

Despite the advancements achieved to date, platelet-rich plasma (PRP) therapy for alopecia continues to present numerous opportunities for enhancement. Future research directions involve proposing solutions for standardizing the protocols, integrating inflammatory markers, and combining PRP with other advanced technologies with the aim of maximizing its clinical efficacy and addressing patients’ needs better.

### 8.1. Standardizing the PRP Protocols

One of the primary challenges remains the absence of a standardized protocol for obtaining and administering PRP. Currently, the centrifugation methods vary considerably in terms of the speed, duration, and component separation techniques. Some research teams prefer double centrifugation to achieve the ideal platelet concentrations (1–1.5 million platelets/μL), while others opt for single centrifugation, adjusting the parameters based on the available equipment [16].

The collection systems used are also diverse, including automated kits such as GPS III or Magellan, which provide high platelet concentrations but also introduce a higher number of leukocytes, potentially problematic in certain pathologies [14]. Other devices, like Selphyl or Arthrex ACP, produce PRP with lower leukocyte and platelet contents, leading to variable outcomes [90]. Regarding the activation methods, the choice between calcium chloride, bovine thrombin, or photostimulation remains debated, with each method influencing growth factor levels (VEGF, TGF-β, PDGF) differently [59,91,92].

Standardizing these variables would have a significant impact, facilitating comparisons of the results across studies and accelerating the development of clear clinical guidelines. Bondarenko and Hausauer and Humphrey have emphasized the importance of uniform terminology, including precise specifications about the type of PRP (leukocyte-poor or leukocyte-rich), platelet concentration, and activation method used [99,100].

### 8.2. Integrating Inflammatory Markers

Another promising direction involves the use of inflammatory markers to predict and monitor the treatment response. Indicators such as the neutrophil–lymphocyte ratio (NLR), the platelet–lymphocyte ratio (PLR), the Systemic Immune-Inflammation Index (SII), and the Systemic Inflammation Response Index (SIRI) have already been validated in other medical fields, and studies suggest their relevance in alopecia [49,50,51,101].

Threshold values for these markers, for example, an NLR between 2.2 and 2.6 or a PLR between 100 and 180, could indicate an inflammatory status conducive to regeneration [102,103]. Furthermore, multifactorial models that integrate these parameters with clinical data, such as hair density as assessed using trichoscopy, could guide therapeutic decisions. Utilizing multi-omics platforms (transcriptomics, proteomics, and metabolomics), future research could identify molecular signatures predictive of treatment success, paving the way for personalized medicine [98].

### 8.3. Combining PRP with Other Technologies

PRP holds significant potential when combined with other therapies, and future research could focus on optimizing these combinations. For instance, the use of PRP alongside low-level laser therapy (LLLT) can stimulate mitochondrial activity and ATP synthesis, enhancing the anagen phase of hair growth. Studies by Gentile and Garcovich have demonstrated that this combination increases hair density and thickness more effectively than PRP administered alone can [80].

Microneedling is another promising technique that facilitates the penetration of growth factors through microchannels created in the stratum corneum. Studies have shown a significant increase in hair density when PRP is combined with this technology [67,87]. Similarly, integrating PRP with active molecules such as minoxidil or finasteride can accelerate regeneration by prolonging the anagen phase and reducing the negative effects of DHT on the hair follicles [82,89].

Anti-inflammatory and nutritional interventions represent another approach with potential. Anti-inflammatory diets and nutraceutical supplements, such as those that reduce levels of IL-1, IL-6, and TNF-α, can create a favorable biological environment for regeneration, enhancing PRP’s efficacy and lowering the risk of recurrence [104,105].

In conclusion, future research directions in the use of PRP for alopecia should focus on three main pillars: standardizing the protocols for PRP preparation and administration; integrating inflammatory markers for patient selection and treatment monitoring; and combining PRP with other innovative technologies. By addressing these objectives, researchers can develop more effective and personalized treatments, offering patients improved outcomes and a better quality of life.

## 9. Discussion

In recent years, hair regeneration has become a major focus in dermatology, with platelet-rich plasma (PRP) therapy emerging as a promising solution for treating alopecia. However, the efficacy of PRP varies considerably among patients, influenced by factors such as the individual’s inflammatory status, the method of PRP preparation, and combinations with other therapeutic interventions. This sections discusses general conclusions about PRP’s potential, the use of biological and inflammatory markers, and the importance of standardizing the protocols and fostering international collaboration initiatives.

### 9.1. The Standard PRP Protocols for Hair Regrowth

In PRP treatment for alopecia, the protocols for PRP’s preparation and administration are pivotal to achieving the optimal outcomes. Typically, blood is drawn into sterile tubes containing 3.2% sodium citrate anticoagulant, which prevents coagulation and preserves the integrity of the PRP [106]. In certain instances, tubes with acid citrate dextrose (ACD) are used to produce leukocyte-rich PRP (L-PRP) via a double centrifugation technique [107]. The centrifugation parameters vary: single centrifugation at 100 g for 10 min yields a moderate platelet concentration, whereas dual centrifugation—such as an initial spin at 160 g followed by a spin at 400 g for 10 min or alternatively a protocol involving a soft spin (1500 rpm for 6 min) and a hard spin (2500 rpm for 15 min)—can result in higher platelet concentrations, although some studies have reported more favorable results using single centrifugation [108,109]. PRP activation is commonly achieved using calcium chloride or via low-temperature activation methods, which modulate the release of the growth factors [106]. Furthermore, the choice of commercial kit—whether a system designed for leukocyte-rich PRP (e.g., Magellan, GPS III) that yields high concentrations of both platelets and leukocytes or one for leukocyte-poor PRP (e.g., Arthrex ACP, Selphyl) that generates a less inflammatory profile—significantly influences therapeutic efficacy [14,91]. The injection techniques include microinjections, network (nappage) injections, and procedures assisted using the Dermapen/microneedling, with the latter demonstrating superior efficacy in enhancing hair density and quality [65,110]. The recommended treatment protocol generally involves 3–4 sessions at intervals of 2–4 weeks, followed by maintenance sessions every 3–6 months [111,112]. Consequently, variations in the collection, centrifugation, activation, and injection techniques underscore the need to tailor the treatment to an individual patient’s specific characteristics.

### 9.2. PRP as a Promising Therapy and the Influence of Inflammatory Status

PRP therapy is recognized for its ability to release essential growth factors—such as VEGF, TGF-β, and FGF—and to reduce local inflammation, thereby contributing to tissue regeneration. Studies have shown that PRP can reactivate the anagen phase of the hair cycle, yielding promising results in conditions like androgenetic alopecia and alopecia areata [26,86].

However, the outcomes are not uniform across all patients. Individuals with an elevated inflammatory status, as indicated by increased values in terms of the NLR, the PLR, or CRP, exhibit a weaker response to treatment. Systemic inflammation appears to interfere with PRP’s beneficial effects, diminishing the capacity of the hair follicles to regenerate hair fibers [86]. Managing inflammation prior to PRP administration is therefore essential to maximize its therapeutic efficacy.

### 9.3. The Use of Inflammatory Markers in Patient Selection

Incorporating inflammatory markers into patient evaluations can facilitate selection and personalize treatment plans. Parameters such as the NLR, PLR, SII, and SIRI have been successfully utilized in other medical fields, and their relevance to alopecia is increasingly being recognized [46,47,49,50,51].

For instance, patients with low NLR and PLR values tend to achieve better outcomes, as a balanced immunological environment favors regeneration. Conversely, elevated levels of these markers may indicate the need for preliminary interventions, such as identifying and treating autoimmune diseases or optimizing nutritional status [46,47,49,50,51]. Additionally, Smith et al. emphasized the importance of measuring CRP to exclude acute inflammations that could influence the response to PRP therapy [113].

Predictive nomograms integrating these markers could be developed to anticipate the likelihood of success with PRP treatment, as demonstrated in other fields like oncology [103].

### 9.4. The Integration of Biological and Growth Markers

Biological factors such as VEGF, TGF-β, and FGF are fundamental to the regenerative effects of PRP. However, pro-inflammatory cytokines like IL-6 and TNF-α can delay or even inhibit the regeneration process. Measuring these biological markers before and after PRP administration provides a detailed perspective on a patient’s therapeutic response.

Different PRP preparation methods—whether leukocyte-poor or leukocyte-rich—also influence its final concentrations of growth factors and cytokines. Studies indicate that PRP prepared through double centrifugation tends to contain higher levels of VEGF and TGF-β, which may enhance its therapeutic efficacy [26,27,28,29]. Integrating multi-omics methodologies, such as transcriptomics and proteomics, can help decipher the interactions among growth factors, cytokines, and patient response, paving the way for personalized medicine [98].

### 9.5. Clarifying the Specificity of Biomarkers

Beyond generalized inflammation and vascular insufficiency, PRP’s efficacy in alopecia is closely tied to its ability to modulate the highly specialized microenvironment of the hair follicles, particularly the bulge region—a niche for the epithelial stem cells critical to hair regeneration. In this context, specific markers such as Cytokeratin 15 (KRT15), Keratin 19 (KRT19), and CD200 can reliably identify regenerative stem cells in the human hair follicle bulge [114,115]. Additionally, markers like CD34 and KRT6 have been identified in murine models to be bulge keratinocyte markers with stem-like properties, demonstrating high proliferative potential and differentiation capacity [116].

Moreover, although inflammatory markers like IL-6 and TNF-α are broadly involved in inflammatory cascades across tissues, their elevated levels in the scalp microenvironment uniquely impair hair follicle function. Increased IL-6 and TNF-α can induce premature catagen entry and suppress the expression of key follicular stem cell genes [58]. In conditions such as lichen planopilaris, when immune privilege in the bulge collapses, T cells infiltrate the area and eradicate KRT15⁺ and CD200⁺ stem cells, underscoring the sensitivity of these markers to inflammation-driven damage [117].

PRP counteracts these detrimental effects by promoting angiogenesis via VEGF, reducing local inflammation through the downregulation of IL-6 and TNF-α, and supporting the viability and proliferation of the bulge stem cells by modulating growth factors such as PDGF, FGF, and IGF. This targeted modulation creates a permissive environment for the reactivation of quiescent KRT15⁺/CD200⁺ bulge cells and facilitates the transition of the follicles into the anagen phase [34,86,118].

Hair loss disorders vary significantly in terms of their pathophysiology, prognosis, and treatment responses. To illustrate, in androgenetic alopecia—a condition driven by DHT-induced follicular miniaturization—PRP therapy has been shown to stimulate follicular activity, increase vascularization, and improve hair density and thickness [66,119]. In alopecia areata, which is characterized by an autoimmune attack on the hair follicles, PRP combines anti-inflammatory action with restoration of immune privilege to induce hair regrowth on par with intralesional corticosteroid treatment, but with a lower risk of side effects [120,121]. Meanwhile, in scarring alopecias—where chronic inflammation and fibrosis lead to irreversible follicular destruction—early-stage PRP treatment may help reduce inflammation and slow the disease progression, although it cannot regenerate destroyed follicles [122]. This evidence underscores the importance of tailoring the treatment approaches to the specific clinical phenotype, and accordingly, we have added a comprehensive discussion of these distinctions to the manuscript.

### 9.6. Expanding on MicroRNAs in PRP: Biological Functions and Biomarker Potential

Recent evidence suggests that microRNAs (miRNAs)—particularly those enriched in platelets and released during PRP activation—play a crucial role in mediating the therapeutic effects of PRP on tissue regeneration, including hair follicle biology. Among the most studied, miR-126 is known for its potent proangiogenic activity, enhancing endothelial cell proliferation and vascular integrity through modulation of the VEGF and FGF signaling pathways [123,124]. This miRNA is actively secreted by activated platelets and contributes significantly to PRP’s regenerative profile. In parallel, miR-21 is highly expressed in PRP and is associated with tissue repair and anti-apoptotic activity, regulating the genes involved in cellular proliferation and inflammation [125]. Notably, both miR-21 and miR-126 have been shown to modulate oxidative stress, immune cell infiltration, and fibrosis in other tissue systems—processes highly relevant to alopecia’s pathogenesis.

Furthermore, circulating levels of platelet-derived miR-126 are responsive to platelet activation and may serve as biomarkers for PRP’s potency and vascular response, as their levels fluctuate with interventions such as aspirin therapy or systemic inflammation [120,126]. In the context of hair regeneration, these miRNAs likely exert epigenetic control over genes regulating the hair cycle, including those linked to follicular angiogenesis, dermal papilla function, and stem cell activation—though this role is still under investigation. As such, miR-21 and miR-126 are emerging not only as active agents within PRP but also as potential biomarkers of the response to PRP therapy in clinical applications like alopecia.

### 9.7. PRP-Derived Extracellular Vesicles (EVs) and Exosomes: Functional Significance and Biomarker Potential

An increasingly recognized component of platelet-rich plasma (PRP) is its content of extracellular vesicles (EVs), particularly exosomes, which act as key mediators of intercellular communication. These nanoscale vesicles (30–150 nm) are enriched with proteins, lipids, and nucleic acids—including regulatory microRNAs (miRNAs) such as miR-126 and miR-21—that contribute directly to angiogenesis, inflammation resolution, and tissue regeneration [127,128]. These exosomal components are thought to complement or even amplify the regenerative effects of soluble platelet-derived growth factors traditionally attributed to PRP.

Importantly, the molecular cargo of these vesicles is protected by a lipid bilayer, making them highly stable and attractive for analysis. Emerging research suggests that the composition of PRP-derived exosomes could serve as a predictive biomarker for PRP’s potency, enabling more personalized and targeted regenerative treatments. For example, quantification of vesicle-associated miRNAs like miR-126 has shown promise in correlating with the angiogenic response and may indicate the therapeutic quality of a given PRP preparation [120]. Nonetheless, this field remains in its early stages, and standardization of the exosome isolation and functional profiling is required before their routine clinical integration.

### 9.8. Proteomic Insights and Biomarker Discovery in PRP Therapy for Alopecia

Recent advances in proteomic technologies have enabled a deeper understanding of the molecular landscape underlying hair loss disorders, identifying key proteins involved in follicular cycling, immune regulation, and tissue remodeling. These findings not only provide insights into pathogenesis but also open avenues for predicting and monitoring the efficacy of treatments like PRP (platelet-rich plasma).

One notable study by Zhang et al. [129] employed TMT-based quantitative proteomics to investigate the changes in protein expression during hair follicle regeneration in mice treated with mesenchymal stem cells. They identified differentially expressed proteins such as KRT25, Stmn1, and Ctps1, all associated with hair follicle cycling and stem cell proliferation—pathways also activated by PRP in human scalp rejuvenation [129].

Similarly, a system-level meta-analysis by Muhammad et al. integrated data from eight transcriptomic datasets, identifying WIF1, SPON1, and KRT35 as differentially expressed proteins linked to Wnt, SHH, and TGF-β signaling—crucial regulators of follicular differentiation and targets modulated by PRP’s growth factor milieu [112].

Moreover, high-throughput proteomic profiling of dermal fibroblasts exposed to different PRP formulations revealed distinct protein signatures between leukocyte-rich PRP and pure PRP, affecting the cell proliferation and inflammatory responses differently [130]. These findings may help explain the inter-patient variability in the clinical outcomes and support the development of personalized PRP protocols based on proteomic screening.

Importantly, such protein expression patterns—particularly those involved in angiogenesis (e.g., VEGF), extracellular matrix remodeling (e.g., MMPs, TIMPs), and oxidative stress—could evolve into biomarkers for the PRP treatment response, allowing clinicians to predict which patients are likely to benefit from therapy or require combination approaches better.

### 9.9. The Necessity of Standardizing the Protocols and Evaluation Methods

The lack of a standardized protocol for PRP preparation and administration remains a major barrier to the widespread validation of this therapy. Variations in the centrifugation parameters, types of kits used, and activation methods lead to heterogeneous results [82,88,89,90].

The assessment of treatment efficacy also suffers from the absence of a rigorous protocol. Methods like trichoscopy, standardized photography, and subjective measurements provide valuable information but are not always applied uniformly [11,48,64]. Adopting common standards—including parameters such as the minimum platelet concentration or the preferred activation method—would facilitate comparisons between studies and contribute to the development of clear clinical guidelines [12,96].

While autologous PRP remains the standard due to its minimal immunological risks, there are specific cases where allogeneic PRP may be considered. For instance, patients who cannot donate sufficient blood—such as those with severe anemia, general frailty, or hematologic disorders—might benefit from an allogeneic product that avoids the additional physiological stress of blood collection [131]. Furthermore, allogeneic PRP derived from healthy donors with an optimal platelet profile could offer a product with standardized, high concentrations of platelets and growth factors, thereby overcoming the variability seen in autologous preparations, particularly in elderly or comorbid patients [132]. In addition, allogeneic PRP facilitates the production of homogeneous batches with a controlled composition and traceability, which is advantageous in research and industrial applications [133]. However, despite promising preliminary data from other clinical fields such as orthopedics and chronic wound management, its application in alopecia is not well established, and extensive testing is necessary to assess its long-term safety, potential immune reactions from repeated administrations, and regulatory considerations similar to those for blood products [134].

### 9.10. International Consensus Initiatives in PRP Use

International collaborations and multicenter studies are essential for validating PRP therapy. Researchers like Llovera and Liesz have demonstrated that scientific validation becomes significantly more robust when protocols are widely tested across multiple centers [94]. Landoni et al. highlighted that many promising results obtained in a single center are not confirmed in multicenter studies, underscoring the need for international consensus initiatives [96].

Adaptive platform models, similar to those used in oncology, could be applied in dermatology to integrate multiple therapies—such as PRP, minoxidil, and low-level laser therapy (LLLT)—and test them simultaneously in a controlled setting [106]. Such initiatives not only reduce the time required to draw conclusions but also ensure more rigorous validation of innovative therapies.

### 9.11. The Pathophysiology of Alopecia Areata and Androgenetic Alopecia and the Importance of Differential Diagnosis

#### 9.11.1. The Pathophysiology of Alopecia Areata

Alopecia areata involves an autoimmune reaction targeting the hair follicles, particularly affecting the bulb and bulge regions. This autoimmune response is characterized by an intense infiltration of lymphocytes, predominantly CD8⁺T cells, which disrupt follicular immune privilege and trigger a premature follicular transition from the anagen to the catagen phase [22,58,118]. Genetic predispositions, psychological stress, and cytokine imbalances, including elevated IL-6 and TNF-α, amplify follicular damage further [25,56,58]. Clinically, alopecia areata presents as patchy, non-scarring hair loss, which may progress to complete scalp involvement (alopecia totalis) or total body hair loss (alopecia universalis), severely affecting patients’ quality of life [1,3,120]. PRP therapy exerts immunomodulatory effects locally by decreasing pro-inflammatory cytokines, thus potentially restoring follicular immune privilege and promoting the reentry of hair follicles into the anagen phase [26,120].

#### 9.11.2. The Pathophysiology of Androgenetic Alopecia

In contrast, androgenetic alopecia (AGA) is predominantly driven by genetic predisposition and androgenic hormones, particularly dihydrotestosterone (DHT). DHT binds to the androgen receptors in dermal papilla cells, resulting in progressive follicular miniaturization, shortening of the anagen phase, and the gradual transformation of terminal hairs into vellus hairs [24,119]. This androgen-mediated process is often exacerbated by perifollicular inflammation and oxidative stress, involving cytokines such as IL-6 and TNF-α, further impairing follicle regeneration [24,58]. PRP counteracts these mechanisms primarily through the release of growth factors like VEGF, PDGF, and FGF, enhancing local vascularization, reducing inflammation, and stimulating dermal papilla proliferation [26,45,119]. However, advanced stages of AGA with severe follicular miniaturization may respond less favorably, underscoring the importance of early therapeutic intervention [81].

#### 9.11.3. Differential Diagnosis and Laboratory Screening

Before initiating PRP treatment, careful consideration of differential diagnoses is essential. One important condition is syphilitic alopecia, which can closely resemble alopecia areata by presenting as patchy, moth-eaten hair loss [68]. Indeed, alopecia may represent an atypical clinical manifestation of secondary syphilis, necessitating serologic screening (e.g., RPR or VDRL tests, confirmed using treponemal-specific assays like FTA-ABS) prior to the initiation of PRP therapy [135]. The failure to recognize syphilitic alopecia may lead to the inappropriate treatment and a delayed diagnosis. Additional conditions to exclude through clinical and laboratory assessments include tinea capitis, trichotillomania, seborrheic dermatitis, and nutritional deficiencies (iron, vitamin D, vitamin B12), as these conditions significantly affect hair growth and PRP outcomes [63].

Integrating these diagnostic considerations into clinical practice ensures the accurate identification of alopecia etiologies, enhances the treatment specificity, and improves overall therapeutic efficacy.

### 9.12. The Limitations and Contraindications of PRP in Alopecia

PRP therapy for alopecia is promising; however, several major limitations must be considered to ensure its proper application and realistic expectations. First, patient responses vary significantly even when PRP is administered correctly, with these differences influenced by systemic inflammatory status (e.g., elevated NLR/PLR values [51]), age, sex, the degree of follicular damage, genetic and immunologic factors, and the presence of comorbidities such as thyroid dysfunction and nutritional deficiencies. Some clinical studies have reported cases where patients showed no improvement despite there being optimal concentrations of growth factors in the PRP [11]. Second, there is a lack of standardization of the PRP preparation protocols, which differ in the volume of blood collected, the type of collection tube and anticoagulant used, the centrifugation method (monospin versus double-spin), and whether activation with CaCl_2_ or thrombin is performed. This variability leads to significant differences in the platelet concentration, the leukocyte content (leukocyte-rich vs. leukocyte-poor PRP), and the final levels of growth factors [14,39]. Third, clear patient selection criteria are lacking; current evidence suggests that PRP may be more effective in early-stage androgenetic alopecia and less beneficial in advanced or cicatricial forms where the follicles are irreversibly damaged. In addition, the therapy is relatively expensive and requires multiple sessions—often 3 to 6 initial treatments, followed by maintenance sessions every 3–6 months—which can be a barrier for some patients. Furthermore, although PRP is minimally invasive, it still involves blood draws and multiple scalp injections that may cause discomfort or anxiety, potentially affecting adherence. Finally, patients with autoimmune diseases, severe nutritional deficiencies, chronic inflammatory conditions, or heavy smoking habits may experience suboptimal responses. In summary, while PRP represents a valuable therapeutic option for alopecia, its efficacy is limited by the individual variability in response, a lack of standardized preparation protocols, high costs, and patient-related factors. Optimizing these variables is essential to achieving consistent and predictable outcomes in clinical practice.

## 10. Conclusions

This review advances the existing knowledge by explicitly emphasizing the importance of systemic inflammation, oxidative stress, and nutritional status as critical factors influencing the effectiveness of platelet-rich plasma (PRP) therapy in alopecia—areas that have received limited attention in the previous literature. It synthesizes evidence supporting the practical use of inflammatory biomarkers, including the neutrophil–lymphocyte ratio (NLR), the platelet–lymphocyte ratio (PLR), IL-6, and TNF-α, as predictors for optimizing individualized PRP treatment. Moreover, it highlights the role of improving antioxidant capacity and correcting deficiencies in vitamins B12, D, and folate to enhance the therapeutic outcomes. By integrating standardized PRP preparation protocols with complementary treatments such as microneedling, minoxidil, and low-level laser therapy, the presented framework offers clinicians a clear and immediately applicable approach. This distinctive emphasis on biomarker-driven patient selection, personalized intervention, and combined modalities provides actionable insights that can significantly improve clinical outcomes in the management of alopecia.

## Data Availability

Not applicable.
